# Gut microbiota and disease

**DOI:** 10.1042/EBC20250046

**Published:** 2026-05-21

**Authors:** Tariq Iqbal

**Affiliations:** 1 Microbiome Treatment Centre, IBR West Link Level, 2.72, University of Birmingham, Birmingham, B15 2TT, U.K.; 2 NIHR Birmingham Biomedical Research Centre, Institute of Translational Medicine, Birmingham, B15 2TH, U.K.

## Abstract

The human gut microbiome—predominantly in the colon—comprises vastly more genetic material than that contained in human cells. Three papers in this journal highlight the functional consequences of imbalance (dysbiosis) of the gut microbiome regarding neurodegenerative conditions, cancer chemotherapy, and the wide-reaching consequences of aberrant metabolism of complex carbohydrates and amino acids in the gut.

The gut is home to a plethora of microbes with the vast majority inhabiting the colon where the community of microbes can amount to 100 billion cells per gram at times [1] and microbial species account for 1000 times more genetic material compared to human genes [2]. This ancient partnership between the microbiome and host provides the human organism with many benefits in terms of immune system programming and regulation, nutrient, and drug metabolism [3]. Our nascent knowledge in this sphere is largely limited to the bacterial content and we know very little about the fungal, archaeal, and viral inhabitants in this space. The human gut microbiome, once established in early life, in spite of the effect of environmental influences such as diet and drugs, remains relatively stable in an individual until senescence [4]. Nevertheless, there is considerable inter-individual variation and this, coupled with our fledgling understanding of the rules governing this biological niche and the thorny problem of a lack of standardisation of analytical methods for assessing the microbiome, results in the fact that, despite a couple of decades of intense genetic sequencing, we remain unable to define what constitutes a normal human microbiome. Nevertheless, we can say that the bacterial community in the gut consists largely of strict obligate anaerobes of the Firmicutes and Bacteriodetes phyla with taxa from Actinobacteria, Proteobacteria, and Verrucomicrobia also contributing [5]. Cross-sectional association studies comparing healthy and diseased individuals have largely led to the concept of ‘dysbiosis’, which is a vague construct of reduced bacterial diversity with a reduction in proportion of ‘beneficial’ commensal anaerobes and expansion in oxygen-tolerant pathobionts. Many association studies have demonstrated dysbiosis in chronic diseases such as Clostridiodes difficile infection (CDI) [6], inflammatory bowel disease (IBD) [7], obesity and metabolic disorders [8], liver diseases [9], cancer [10], and neurodegenerative diseases [11]. There are some indications regarding the importance of key organisms for example reduction in Faecalobacterium prausnitzii in Crohn’s disease [12], overrepresentation of Prevotella and Ruminococcus in (Western) obesity related metabolic disease [13], and Enterococcus faecalis in alcoholic liver disease [14]. The clearest evidence of dysbiosis in relation to chronic disease comes from IBD. A recent metanalysis of data from inception IBD patients has shown a reduced diversity and an over-abundance of oral pathobionts in IBD compared to healthy subjects [15]. However, it is difficult to draw clear conclusions from these associations because of a lack of standardised methodology in this fast-evolving field.

Despite clear signs of dysbiosis, interest in microbiome manipulation as a treatment has really come from the remarkable success of ‘whole community’ replacement attempts using fecal microbiota transplant (FMT) in the treatment of CDI [16]. FMT involves the transfer of minimally processed fecal samples from screened healthy donors to patients. Indeed, this has now become standard of care for recurrent CDI. Although FMT is problematic due to donor dependence, difficulties in production at scale, variability of the end product, ignorance of the underlying mechanisms of action, and the potential for pathogen transmission [17], on the basis of ‘it seems to work’ it has been trialed to good effect in IBD [18]. However, if we are to move to reliable microbial treatments for disease, a robust understanding of underlying mechanisms is an urgent necessity.

In this regard, focus on the metabolites produced by the microbiome is key and, in recent years, a large body of work focusing on the metabolites generated by gut microbiota has begun to clear the fog in key aspects [19]. We rely on gut microbiota for the fermentation of complex carbohydrates and some amino acids. Short-chain fatty acids resulting from this fermentation are powerful agents mediating local and systemic immune responses [20] and epithelial integrity [19]. The effect of the gut microbiota in generating secondary bile acids similarly drives an immune response [21]. In the context of IBD the production of indole derivatives by the action of microbiota on the essential amino acid tryptophan leads to an anti-inflammatory immune response and epithelial barrier enhancement via the activation of the transcription factor aryl hydrocarbon receptor AhR downsteam [22,23].

This journal contains three important updates concerning metabolomic consequences of microbiome dysbiosis. In the first paper, Park et al. outline microbiome dysbiosis associated with Alzheimer’s disease and emerging data with respect to associated metabolite and neurotransmitter changes and indeed direct bacterial translocation across the blood brain barrier associated with inflammation [24]. While largely based on animal models such experiments seem to have great potential in our developing understanding of this devastating illness. Berretta and Schwab provide a detailed and up to date review of the consequences of carbohydrate and amino acid fermentation in the gut highlighting short-chain fatty acid metabolism in various anatomical sections of the gut and the physiological consequences of this [25]. Finally, Dormans et al. consider the important emerging actions of tryptophan metabolites in mediating carcinogenesis, potential action in enhancing the effects of immune checkpoint inhibitor treatment and in the pathogenesis of atherosclerosis [26].

Currently our understanding of the complex interplay between microbes in the gut and the physiological consequences of this is very much in its infancy [17]. In order to move from association studies and FMT trials towards rational microbiome modulation in the treatment of disease, it is imperative that deeper understanding of the functional impacts associated with microbiota changes observed in disease is developed following the approaches described in these papers.

## Open Access

Open access for this article was enabled by the participation of the University of Birmingham in an all-inclusive Read & Publish agreement with Portland Press and the Biochemical Society under an agreement with JISC.

## Abbreviations

CDI: Clostridiodes difficile infection
FMT: Fecal microbiota transplant
IBD: Inflammatory bowel disease
